# Prognostic value of post-operative serum procalcitonin in gastric adenocarcinoma patients undergoing radical gastrectomy: propensity score matching analysis of extended cohort from a prospective bi-center study

**DOI:** 10.1007/s10120-023-01422-0

**Published:** 2023-08-14

**Authors:** Hua Xiao, Yongzhou Huang, Peng Zhang, Huijun Zhou, Dian Liu, Jia Luo

**Affiliations:** 1 Department of Hepatobiliary and Intestinal Surgery, Hunan Cancer Hospital and The Affiliated Cancer Hospital of Xiangya School of Medicine, Central South University, 283 Tongzipo Road, Changsha, 410013 Hunan China; 2 Department of Gastroduodenal and Pancreatic Surgery, Hunan Cancer Hospital and The Affiliated Cancer Hospital of Xiangya School of Medicine, Central South University, Changsha, 410013 China; 3 Department of Gastrointestinal Surgery, Union Hospital, Tongji Medical College, Huazhong University of Science and Technology, Wuhan, 430022 China; 4 Department of General Surgery, First Affiliated Hospital, School of Medicine, Shihezi University, Shihezi, 832008 China; 5 Department of Gastroenterology and Urology, Hunan Cancer Hospital and The Affiliated Cancer Hospital of Xiangya School of Medicine, Central South University, Changsha, 410013 China; 6 Department of Lamphoma and Abdominal Radiotherapy, Hunan Cancer Hospital and The Affiliated Cancer Hospital of Xiangya School of Medicine, Central South University, Changsha, 410013 China

**Keywords:** Gastric adenocarcinoma, Gastrectomy, Procalcitonin, Prognosis, Validation, Propensity score matching analysis

## Abstract

**Background:**

The aim of this study was to investigate the predictive value of procalcitonin (PCT) on post-operative day (POD) 3 and 5 for the prognosis of gastric adenocarcinoma (GA) patients who underwent radical gastrectomy surgery in extended cohort from a prospective bi-center study.

**Methods:**

Consecutive GA patients who received surgery in the Hunan Cancer Hospital were enrolled as the training cohort, and those from Wuhan Union Hospital were included as external validation cohort. The optimal cutoff concentration of PCT for overall survival (OS) in the training cohort was determined by X-tile. The independent predictive factors for OS were identified using univariate and multivariate Cox regression analyses. Furthermore, the predictive value of elevated PCT was clarified in the validation cohort and propensity score matched cohort, respectively.

**Results:**

The optimal cutoff concentrations of PCT for OS were 0.67 ng/mL at POD 3 and 0.39 ng/mL at POD 5 in the training cohort (n = 906). Patients with higher PCT concentrations (≥ 0.39 ng/mL) at POD 5 had a significantly worse prognosis whether developing post-operative infections or not. Moreover, a synergistic influence was confirmed in those with elevated PCT concentration and infections. Multivariate analyses confirmed that PCT concentration ≥ 0.39 ng/mL at POD 5 was significantly associated with poorer survival in training cohort (HR: 1.422, 95% CI 1.041–1.943, *P* = 0.027), validation cohort (n = 297, HR: 2.136, 95% CI 1.073–4.252, *P* = 0.031) and matched cohort (n = 901, HR: 1.454, 95% CI 1.104–1.914, *P* = 0.008), separately.

**Conclusions:**

PCT concentration ≥ 0.39 ng/mL at POD 5 was a reliable predictor for poorer prognosis in GA patients undergoing radical gastrectomy.

**Supplementary Information:**

The online version contains supplementary material available at 10.1007/s10120-023-01422-0.

## Introduction

Gastric adenocarcinoma (GA), with an estimated incidence of over one million new cases in 2020 worldwide, is one of the most common malignancies, with gastrectomy offering the only possible curative treatment to date [1, 2]. Unfortunately, the long-term outcomes of GA patients are still dismal given that only about 30% of patients are diagnosed at an early stage in the West and in China. Although the pathological tumor, node and metastasis (pTNM) stage have been the most widely used factors to predict the prognosis of malignancies, there has been increasing evidence that nutritional and inflammatory statuses are significantly associated with the oncological outcomes of numerous cancers. Several systemic inflammation-based variables have been confirmed to influence survival of GA patients, such as lymphocyte count, C-reactive protein (CRP), the neutrophil to lymphocyte ratio (NLR), Glasgow Prognostic Score (GPS), prognostic nutritional index (PNI), etc. [3–7].

Procalcitonin (PCT), the precursor of calcitonin consisting of 116 amino acids, has been well established as an inflammatory indicator [8–10]. In order to clarify the predictive value of PCT for post-operative infection following gastrectomy in GA patients, we conducted a prospective study in two high-volume tertiary hospitals in China from June 2018 to July 2019 (ClinicalTrails.gov number: NCT03780439). A total of 552 patients were enrolled in the study and our research confirmed that PCT was a more accurate marker than white blood and neutrophil counts to indicate infection [11]. Since then, PCT concentrations have been routinely measured at post-operative day (POD) 3 and POD 5 in patients undergoing gastrectomy in our centers.

In recent years, more and more studies have reported that various types of cancers secret PCT and that PCT is a reliable predictor for long term survival, including for medullary thyroid cancer, colorectal cancer, non-small cell lung cancer (NSCLC), and others [12–15]. But whether the serum concentration of PCT was related to the oncological outcomes of GA patients has never been investigated. Therefore, for the first time, we conducted this study to clarify whether the serum PCT concentration could predict the prognosis of GA patients following radical gastrectomy, using retrospectively collected data obtained from a large cohort of patients in two tertiary hospitals in China.

## Methods

### Study design

**Fig. 1:**
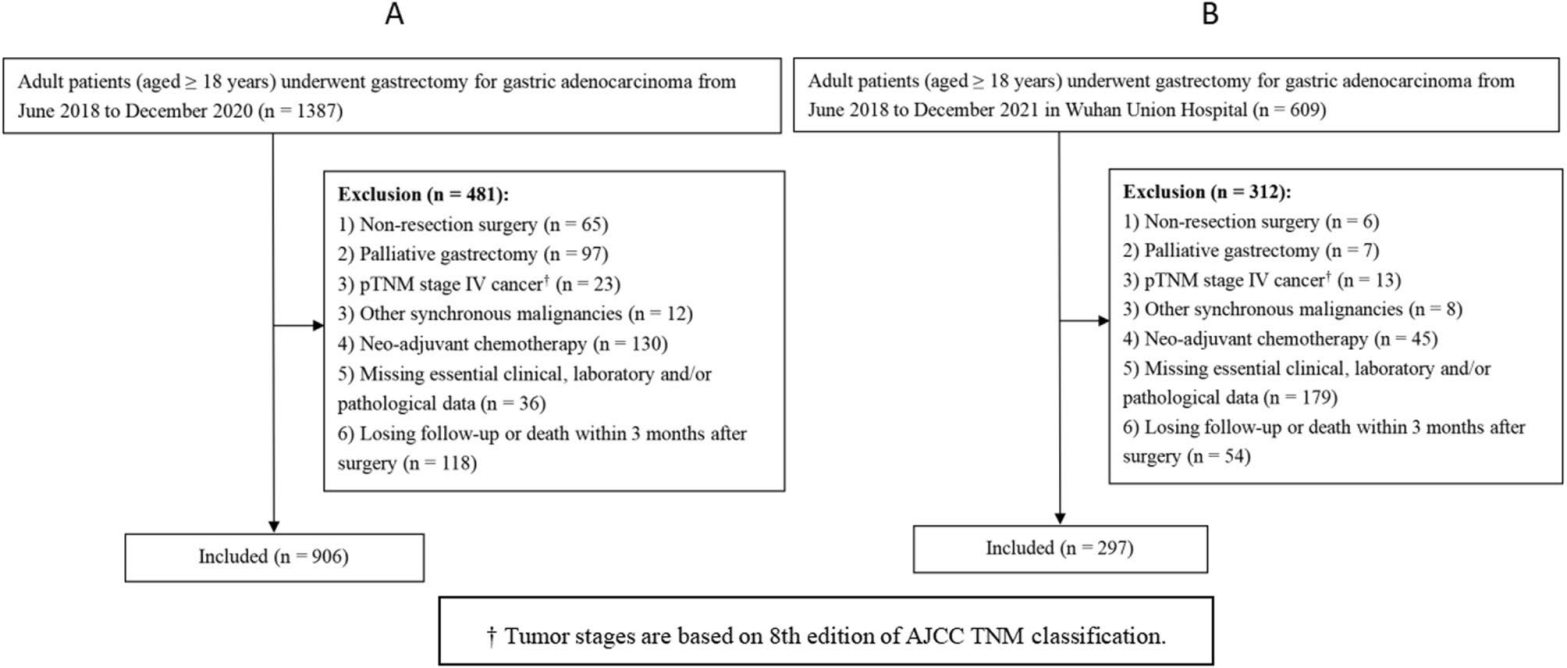
Flow diagram of the training cohort **A** and the validation cohort **B** in the present study

The medical records of consecutive patients with pathologically confirmed GA who underwent operations in Hunan Cancer Hospital from June 2018 to November 2020 and in Wuhan Union Hospital from June 2018 to November 2021 were reviewed. The demographic and clinicopathological data, operative variables and follow-up data were prospectively collected and retrospectively analyzed. The inclusion and exclusion criteria for this study and the flowchart of patients are shown in Fig. 1. Patients from Hunan Cancer Hospital were enrolled into the training cohort (Fig. 1A), whereas those from Wuhan Union Hospital following the same inclusion and exclusion criteria were included as the validation cohort (Fig. 1B). This study was approved by the ethics committee of our institutions (No. 41 in 2023) and written informed consent was obtained from all enrolled patients.

### Peri-operative management and follow-up

Surgeons with sufficient experience performed or supervised all operations in line with the guidelines and stages according to the eighth edition of the TNM system [16–18]. A second- or third-generation cephalosporin was given as a prophylactic antibiotic lasting for about 72 h to all patients. From June 2018, the serum PCT concentration has been routinely measured at POD 3 and 5 in patients who underwent gastrectomy for GA in our centers, using an automatic electrochemiluminescence immunoassay analyzer as described in our previous study [11]. The normal reference range of PCT concentrations was < 0.050 ng/mL. The prognostic nutritional index (PNI) was calculated as serum albumin level (g/L) + 0.005 × total lymphocyte count in the peripheral blood (per mm^3^) [6]. In line with previous studies [19, 20], a PNI value of at least 50 was defined as normal, with values between 40 and 50 regarded as mild-moderate malnutrition, and values lower than 40 as serious malnutrition. Neutrophil-to-lymphocyte ratio (NLR) was calculated as dividing neutrophil count by lymphocyte count in the peripheral blood.

Post-operative complications were diagnosed and staged according to the Clavien-Dindo system and only grade II or greater morbidities were analyzed [21]. Post-operative infections, such as intra-abdominal infection, pulmonary infection, wound infection, sepsis, urinary tract infection and catheter-related infections, was diagnosed according to the definition of the Centers for Disease Control and Prevention [22] and briefly described in our previous study [11]. Patients with pTNM stage II/III GC were recommended to receive adjuvant chemotherapy about 4 weeks after surgery and were treated with fluorouracil- and platinum-based regimens, such as XELOX and SOX combinations [23–25].

Patients were followed-up every 3 months by outpatient review or telephone in the first 2 years and then every 6 months from the 3rd to 5th year and once a year thereafter, until February 2023. Overall survival (OS) was calculated as the month from surgery to death or the last follow-up time, whichever occurred first.

## Statistical analysis

A χ^2^ test, Fisher’s exact test or a Student’s *t*-test were employed to evaluate potential differences between categorical and continuous variables when appropriate. The optimal cut-off concentration of PCT for OS was affirmed by X-tile (3.6.1 software 20, http://medicine.yale.edu/lab/rimm/research/software.aspx) when it reached the maximum χ2 log-rank value, as described in previous studies [4, 6]. Potential predictive factors for OS were first investigated using univariate analysis. Variables with a *P*-value < 0.05 in the univariate analysis were further enrolled into multivariate Cox regression analyses. Kaplan–Meier curves and log-rank tests were applied to compare the OS of patients in different groups. To minimize the potential influence of selective bias, patients with elevated PCT concentrations were matched to those with relatively low PCT concentration using a propensity score as previously described [26, 27]. Nearest neighbor matching with a caliper width of a 0.05 standard deviation was performed at a 1:3 ratio without replacement. Data analyses were performed using R version 4.0.1 and a correlated package or SPSS version 27.0. A two-tailed *P*-value < 0.05 was considered to be statistically significant.

## Results

### Characteristics of patients

As shown in Table 1, 906 consecutive patients from the Hunan Cancer Hospital were enrolled into the training cohort and another 297 consecutive patients from the Wuhan Union Hospital were included as the validation cohort. Among the entire 1203 patients, the mean age was 57.59 years (range 22–84), with a mean body mass index (BMI) of 22.28 kg/m^2^ (range 14.53–37.13), and a mean post-operative hospital stay of 10.35 days (range 4–87). More than half of the patients were male (64.6%), who underwent distal subtotal gastrectomy (72.4%) by a laparoscopic procedure (59.2%). In line with the 8th version of the staging system, there were 377 (31.3%) stage I, 278 (23.1%) stage II and 548 (45.6%) stage III cases. The mean surgical time was 221.35 min (range, 55–640), and the mean intra-operative bleeding was 175.53 mL (range, 20–2000). A total of 189 patients (15.7%) developed grade II or greater post-operative complications and there were 87 cases (7.2%) of infection.

**Table 1:** Clinicopathological characteristics of the training and validation cohort of patients with stage I-III gastric cancer (n = 1203)

Variables	The entire cohort (n = 1203)	Training group (n = 906)	Validation group (n = 297)`	*P* value
Gender (males)	777 (64.6%)	585 (64.6%)	192 (64.6%)	0.981
Age (years)	57.59 ± 10.76	57.52 ± 10.80	57.83 ± 10.64	0.666
Body Mass Index (kg/m^2^)	22.28 ± 3.14	22.19 ± 3.10	22.55 ± 3.25	0.086
Any comorbidities	295 (24.5%)	254 (28.0%)	41 (13.8%)	< 0.001
ASA score				< 0.001
1	127 (10.6%)	45 (5.0%)	82 (27.6%)	
2	1043 (86.7%)	841 (92.8%)	202 (68.0%)	
3	33 (2.7%)	20 (2.2%)	13 (4.4%)	
Pre-operative lymphocyte count (× 10^9^/L)	1.94 ± 0.91	1.79 ± 0.67	2.39 ± 1.31	< 0.001
Pre-operative albumin concentration (g/L)	41.11 ± 4.47	41.79 ± 4.29	39.08 ± 4.30	< 0.001
Pre-operative hemoglobin (g/L)	122.65 ± 24.92	123.20 ± 25.80	120.95 ± 21.99	0.176
Operation method				< 0.001
Open	491 (40.8%)	458 (50.6%)	33 (11.11%)	
Laparoscopy	712 (59.2%)	448 (49.4%)	264 (88.89%)	
Type of resection				< 0.001
Distal subtotal gastrectomy	871 (72.4%)	704 (77.7%)	167 (56.2%)	
Proximal subtotal gastrectomy	35 (2.9%)	12 (1.3%)	23 (7.7%)	
Total gastrectomy	297 (24.7%)	190 (21.0%)	107 (36.0%)	
Lymph node harvested	21.69 ± 9.13	20.06 ± 7.74	26.64 ± 11.09	< 0.001
T stage*				0.002
T1	309 (25.7%)	215 (23.7%)	94 (31.6%)	
T2	179 (14.9%)	137 (15.1%)	42 (14.1%)	
T3	275 (22.9%)	198 (21.9%)	77 (25.9%)	
T4	440 (36.6%)	356 (39.9%)	84 (28.3%)	
N stage*				0.035
N0	498 (41.4%)	354 (39.1%)	144 (48.5%)	
N1	193 (16.0%)	149 (16.4%)	44 (14.8%)	
N2	204 (17.0%)	158 (17.4%)	46 (15.5%)	
N3	308 (25.6%)	245 (27.0%)	63 (21.2%)	
pTNM stage*				< 0.001
I	377 (31.3%)	262 (28.9%)	115 (38.7%)	
II	278 (23.1%)	201 (22.2%)	77 (25.9%)	
III	548 (45.6%)	443 (48.9%)	105 (35.4%)	
Intra-operative blood loss (mL)	175.53 ± 174.67	200.66 ± 176.12	98.89 ± 145.69	< 0.001
Operation time (min)	221.35 ± 79.75	198.95 ± 62.22	289.71 ± 88.13	< 0.001
Post-operative complications†				< 0.001
None	1014 (84.3%)	792 (87.4%)	222 (74.7%)	
Infectious complications	87 (7.2%)	51 (5.6%)	36 (12.1%)	
None-infectious complications	102 (8.5%)	63 (7.0%)	39 (13.1%)	
Post-operative hospital stays (days)	10.35 ± 5.17	10.00 ± 5.04	11.43 ± 5.42	< 0.001
Procalcitonin level at POD 3 (ng/mL)	0.87 ± 3.37	0.49 ± 0.98	2.02 ± 6.42	< 0.001
Procalcitonin level at POD 5 (ng/mL)	0.48 ± 3.01	0.41 ± 3.34	0.70 ± 1.58	0.156
Adjuvant chemotherapy				0.554
No	683 (56.8%)	510 (56.3%)	173 (58.2%)	
Yes	520 (43.2%)	396 (43.7%)	124 (41.8%)	

Data are presented as mean ± SD or n (%). ASA, American Society of Anesthesiologists. POD, post-operative day

^*^ Tumor stages are based on 8th edition of the Union for International Cancer Control TNM classification

^†^ Defined as Clavien-Dindo grade II or greater. Patients who developed both infectious and non-infectious complications were classified into the infectious group

As shown in Table 1, patients in the validation group had significantly lower ratios of comorbidities, lesser American Society of Anesthesiologists (ASA) scores, lower concentrations of pre-operative albumin and less intra-operative blood loss compared to the training group. But more patients with early-stage GA who underwent laparoscopic surgery, developed post-operative complications, had longer operation times and post-operative hospital stays (all *P* < 0.05). In addition, the PCT concentration at POD 3 was significantly higher in the validation group, but comparable at POD 5.

### Predictors for OS in the training cohort

With a median follow-up time of 31.0 months (range, 4–58), neither the median recurrence free survival (RFS) time nor the median OS time were reached in the training cohort. A total of 251 cases (27.7%) of recurrence and 231 cases (25.5%) of death were recorded, with 1-, 2-, 3- and 4-year OS rates of 92.3%, 80.5%, 75.0% and 68.2%, respectively. The cut-off concentrations of PCT at POD 3 and POD 5 for OS were set at 0.67 ng/mL and 0.39 ng/mL by X-tile, when they reached the maximum χ^2^ values of 8.8624 and 14.3769, with the minimum log-rank test *P-*value of 0.0604 and 0.0050, separately (Supplementary Fig. 1). Similarly, the best cutoff value of NLR was set as 2.84 by X-tile, when it reached the maximum χ^2^ value of 17.4698 and minimum log-rank test *P-*value of 0.0012. As presented in Table 2, univariate analyses revealed that age, the hemoglobin and albumin concentrations, lymphocyte count, PNI, NLR, intra-operative blood loss, tumor stage, peri-operative blood transfusion, post-operative complication, PCT concentrations at POD 3 and POD 5 were potentially associated with OS (all *P* < 0.05). Further multivariate Cox regression analyses containing the above mentioned factors confirmed that older age (≥ 65 years), larger intra-operative blood loss (≥ 300 mL), advanced tumor stage, post-operative complication and higher PCT concentrations (≥ 0.39 ng/mL) at POD 5 were independent predictors for unsatisfactory oncological outcomes.

**Table 2:** Univariate and multivariate analyses of prognostic factors for overall survival following radical gastrectomy of gastric adenocarcinoma in the training cohort (n = 906)

Variables	N	3-year overall survival rate (%)	UV *P* value	MV HR (95% CI)	MV *P* value
Gender			0.430		
Male	585	75.6			
Female	321	73.7			
Age (years)			0.003		0.042
< 65	645	77.7		Reference	
≥ 65	261	68.1		1.331 (1.011–1.752)	
Body mass index (kg/m^2^)			0.243		
≥ 18.5	800	75.9			
< 18.5	106	66.8			
Comorbidities			0.405		
Yes	254	76.4			
No	652	74.3			
Hemoglobin (g/L)			0.002		0.900
≥ 100	720	77.6			
< 100	186	64.0			
Albumin level (g/L)			0.001		0.730
≥ 35	845	76.3			
< 35	60	55.2			
Lymphocyte count (× 10^9^/L)			0.006		0.394
≥ 1.5	611	77.3			
< 1.5	295	69.7			
Prognostic nutritional index			< 0.001		0.405
> 50	520	79.9			
40–50	351	68.7			
< 40	35	51.8			
Neutrophil-to-lymphocyte ratio			< 0.001		0.172
< 2.84	731	77.5			
≥ 2.84	175	63.6			
Intra-operative blood loss (mL)			< 0.001		0.032
< 300	751	76.9		Reference	
≥ 300	154	65.0		1.405 (1.029–1.918)	
pTNM stage ^†^			< 0.001		
I	262	94.7		Reference	
II	201	86.8		2.587 (1.323–5.058)	0.005
III	443	57.0		10.369 (5.907–18.201)	< 0.001
Peri-operative blood transfusion			< 0.001		0.444
No	746	78.1			
Yes	160	59.4			
Post-operative complication^‡^			< 0.001		0.007
No	792	76.7		Reference	
Yes	114	58.8		1.593 (1.137–2.231)	
Procalcitonin level at post-operative day 3 (ng/mL)			0.002		0.981
< 0.67	771	76.6			
≥ 0.67	135	64.7			
Procalcitonin level at post-operative day 5 (ng/mL)			< 0.001		0.027
< 0.39	775	76.9		Reference	
≥ 0.39	131	63.0		1.422 (1.041–1.943)	

CI, confidence interval; HR, hazard ratio; UV, univariate analysis; MV, multivariate analysis

^†^ Tumor stages are based on 8th edition of AJCC TNM classification

^‡^ Defined as Clavien-Dindo grade II or greater

### Association among PCT, infection and prognosis in the training cohort

**Fig. 2:**
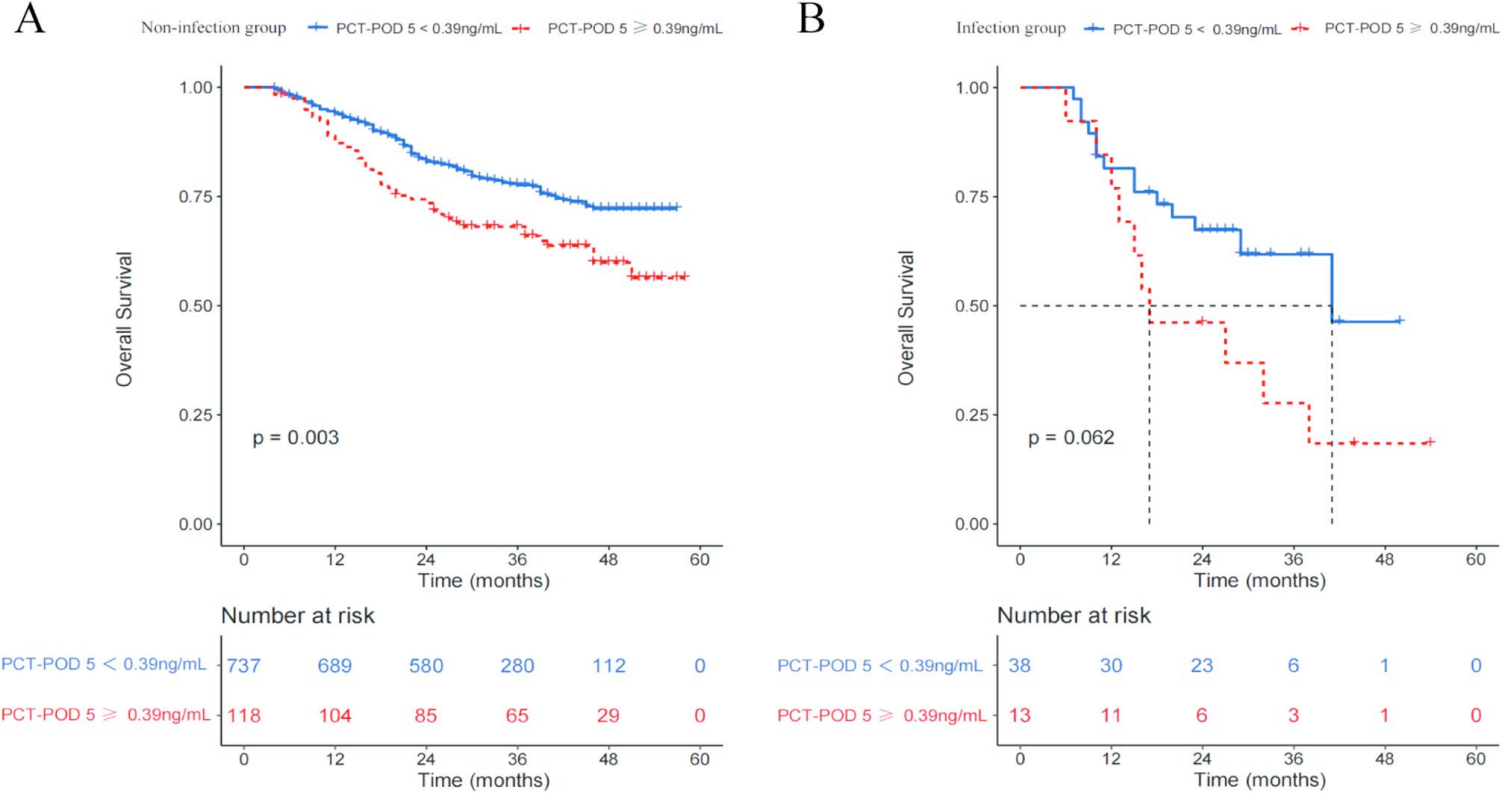
Over survival curves of the 906 patients in the training cohort who underwent curative resection for stage I-III gastric cancer stratified by procalcitonin (PCT) level at post-operative day (POD) 5 (< 0.39 or ≥ 0.39 ng/mL) in the non-infection group **A** and infection group **B**

To explore the association with PCT concentrations, post-operative infection and prognosis in the training cohort, we further classified the 906 patients into infection (n = 51, 5.6%) and non-infection (n = 855, 94.4%) subgroups. As shown in Fig. 2, non-infection patients with higher PCT concentrations at POD 5 were associated with significantly more unfavorable OS than those with lower PCT concentrations (*P* = 0.003). Similarly, patients with infections and higher PCT concentrations seemed to have worse prognosis, but the difference did not reach statistical significance (*P* = 0.062).

In addition, to investigate the potential additive impact of an increased PCT concentration and infection on survival, we further stratified the 906 patients into 4 subgroups based on whether the PCT concentration was ≥ 0.39 ng/mL at POD 5 or the patient was infected or not: PCT low/infection (−) (n = 737), PCT high/infection (−) (n = 118), PCT low/infection ( +) (n = 38) and PCT high/infection ( +) (n = 13), respectively. Not surprisingly, patients with elevated PCT concentrations and infections had the worst oncological outcomes (Table 3 and Supplementary Fig. 2). A synergistic influence was confirmed in the PCT high/infection ( +) group comparing to the PCT high/infection (−) group (hazard ratio [HR]: 2.916, 95% confidence interval [CI] 1.460–5.824, *P* = 0.002).

**Table 3:** Subgroup analysis of overall survival (OS) of stage I-III gastric adenocarcinoma following radical gastrectomy in the training cohort (n = 906)

Subgroups	n (%)	3-year OS rate %	Hazard Ratio (HR)	95% Confidence Interval [CI]	P value
PCT low/infection (−)	737 (81.3%)	77.6	Reference	Reference	
PCT high/infection (−)	118 (13.0%)	66.5	1.645	1.178–2.297	0.003
PCT low/infection ( +)	38 (4.2%)	52.4	2.198	1.272–3.798	0.005
PCT high/infection ( +)	13 (1.4%)	21.7	4.968	2.621–9.416	< 0.001

PCT, procalcitonin; OS, overall survival

PCT low was defined as < 0.39 ng/mL at post-operative day 5, PCT high was defined as ≥ 0.39 ng/mL at post-operative day 5

Infection ( +) and infection(−) was defined as developing post-operative infectious complications or not

### Oncological outcomes in the validation cohort

Among the 297 patients in the validation group, 23 experienced recurrence (7.7%) and 43 (14.5%) died with a median follow-up of 25.0 months (range, 6–56). The 1-, 2-, 3- and 4-year OS rates were 98.3%, 93.1%, 80.9% and 70.1%, separately. Given the relatively short duration of the follow-up time, neither the median RFS time nor the median OS time could be determined. As presented in Supplementary Table 1, univariate analyses revealed that age, BMI, tumor stage and the PCT concentration at POD 3 and POD 5 were potentially predictors for OS (all *P* < 0.05). After using these 5 variables in further multivariate Cox regression analyses, a higher PCT concentration (≥ 0.39 ng/mL) at POD 5 along with an older age (≥ 65 years), lower BMI (< 18.5 kg/m^2^) and advanced tumor stage were confirmed to be independently related to OS.

### Propensity score matching (PSM) analysis

Propensity scores were conducted using variables such as age, BMI, intra-operative blood loss, post-operative complications and the TNM stage, which had been confirmed as significant predictors for OS in the training and/or validation group. The matched cohort was comprised of 901 patients after one-to-three PSM, with 250 patients in the PCT high concentration group (≥ 0.39 ng/mL at POD 5) and 651 in the PCT low concentration group (< 0.39 ng/mL at POD 5), respectively. As described in Table 4, the baseline characteristics such as gender, age, BMI, post-operative complications and TNM stage differed significantly before matching, but became comparable after matching. Further univariate and multivariate Cox regression analyses also confirmed that a PCT concentration ≥ 0.39 ng/mL at POD 5 was significantly associated with OS in the matched cohort (HR: 1.454, 95% CI 1.104–1.914, *P* = 0.008, Table 5). As shown in Supplementary Fig. 3, patients with elevated PCT concentrations at POD 5 had significantly worse oncological outcomes no matter before and after matching (*P* < 0.001 and *P* = 0.033, respectively).

**Table 4:** Clinicopathological characteristics of the entire study cohort stratified by procalcitonin (PCT) level at post-operative day 5, before and after propensity score matching

Variables	Total cohort (n = 1203)			Propensity score matched cohort (n = 901)		
	PCT ≥ 0.39 ng/mL group (n = 270)	PCT < 0.39 ng/mL group (n = 933)	*P* value	PCT ≥ 0.39 ng/mL group (n = 250)	PCT < 0.39 ng/mL group (n = 651)	*P* value
Gender (males)	201 (74.4%)	576 (61.7%)	< 0.001	182 (72.8%)	459 (70.5%)	0.496
Age (years)	60.47 ± 10.93	56.76 ± 10.58	< 0.001	59.70 ± 10.89	58.28 ± 10.77	0.076
Body Mass Index (kg/m^2^)	21.64 ± 3.07	22.46 ± 3.14	< 0.001	21.94 ± 3.09	22.35 ± 3.19	0.079
Any comorbidities	68 (25.2%)	227 (24.3%)	0.774	62 (24.8%)	162 (24.9%)	0.979
Pre-operative lymphocyte count (× 10^9^/L)	1.98 ± 0.97	1.93 ± 0.90	0.351	1.99 ± 0.96	1.92 ± 0.94	0.391
Intra-operative blood loss (mL)	159.69 ± 194.69	180.12 ± 168.27	0.091	160.74 ± 196.96	182.97 ± 174.02	0.099
Post-operative complications^†^			< 0.001			0.464
None	205 (75.9%)	809 (86.7%)		205 (82.0%)	547 (84.0%)	
Yes	65 (24.1%)	124 (13.1%)		45 (18.0%)	104 (16.0%)	
T stage*			< 0.001			0.280
T1	44 (16.3%)	265 (28.4%)		42 (16.8%)	147 (22.6%)	
T2	41 (15.2%)	138 (14.8%)		38 (15.2%)	93 (14.3%)	
T3	70 (25.9%)	205 (22.0%)		64 (25.6%)	147 (22.6%)	
T4	115 (42.6%)	325 (34.8%)		106 (42.4%)	264 (40.6%)	
N stage*			0.411			0.876
N0	102 (37.8%)	396 (42.4%)		94 (37.6%)	246 (37.8%)	
N1	46 (17.0%)	147 (15.8%)		42 (16.8%)	101 (15.5%)	
N2	44 (16.3%)	160 (17.1%)		42 (16.8%)	123 (18.9%)	
N3	78 (28.9%)	230 (24.7%)		72 (28.8%)	181 (27.8%)	
pTNM stage*			0.021			0.134
I	66 (24.5%)	311 (33.3%)		61 (24.4%)	180 (27.6%)	
II	69 (25.6%)	209 (22.4%)		65 (26.0%)	130 (20.0%)	
III	135 (50.0%)	413 (44.3%)		124 (49.6%)	341 (52.4%)	
Post-operative hospital stays (days)	10.00 ± 5.04	11.43 ± 5.42	< 0.001	10.31 ± 3.91	10.46 ± 5.71	0.703
Adjuvant chemotherapy (yes)	112 (41.5%)	408 (43.7%)	0.511	105 (42.0%)	290 (44.5%)	0.490

Data are presented as mean ± SD or n (%)

^†^ Defined as Clavien-Dindo grade II or greater

^*^Tumor stages are based on 8th edition of the Union for International Cancer Control TNM classification

**Table 5:** Univariate and multivariate analyses of prognostic factors for overall survival following radical gastrectomy of gastric adenocarcinoma in the propensity score matched cohort (n = 901)

Variables	N	3-year overall survival rate (%)	UV *P* value	MV HR (95% CI)	MV *P* value
Gender			0.050		0.105
Male	641	75.2			
Female	260	68.6			
Age (years)			0.017		0.022
< 65	610	76.2		Reference	
≥ 65	291	67.3		1.368 (1.045–1.790)	
Body mass index (kg/m^2^)			0.137		
≥ 18.5	783	74.6			
< 18.5	118	64.8			
Comorbidities			0.861		
Yes	224	73.4			
No	677	73.2			
Hemoglobin (g/L)			0.018		0.834
≥ 100	705	75.8			
< 100	196	63.7			
Albumin level (g/L)			0.005		0.314
≥ 35	815	74.7			
< 35	86	56.9			
Lymphocyte count (× 10^9^/L)			< 0.001		0.038
≥ 1.5	621	76.3		Reference	
< 1.5	280	65.9		1.332 (1.017–1.746)	
Intra-operative blood loss (mL)			0.013		
< 300	769	75.7			
≥ 300	132	65.2			
pTNM stage ^†^			< 0.001		< 0.001
I	241	92.1		Reference	
II	195	88.0		1.986 (1.059–3.726)	
III	465	57.6		7.642 (4.476–12.439)	
Post-operative complication^‡^			0.034		0.118
No	752	74.9			
Yes	149	65.9			
Procalcitonin level at post-operative day 3 (ng/mL)			0.837		
< 0.67	593	73.9			
≥ 0.67	308	72.4			
Procalcitonin level at post-operative day 5 (ng/mL)			0.033		0.008
< 0.39	651	75.3		Reference	
≥ 0.39	250	68.8		1.454 (1.104–1.914)	

CI, confidence interval; HR, hazard ratio; UV, univariate analysis; MV, multivariate analysis

^†^ Tumor stages are based on 8th edition of AJCC TNM classification

^‡^ Defined as Clavien-Dindo grade II or greater

## Discussion

In this post hoc analysis with an extended cohort from our previously prospective study, for the first time it has been revealed that a higher serum PCT concentration (≥ 0.39 ng/mL) at POD 5 was an independent predictor for unfavourable prognosis in stage I-III GA patients who underwent curative resection. Subgroup analyses confirmed that an increased PCT concentration significantly adversely impacted the prognosis in patients no matter whether they developed post-operative infections or not. In addition, a synergistic effect was shown given that patients with elevated PCT concentrations and suffering from infection had an even worse prognosis, compared to those with elevated PCT concentrations but non-infected (HR: 2.916, *P* = 0.002). In the external validation cohort of 297 patients, a PCT concentration ≥ 0.39 ng/mL at POD 5 was also confirmed to be significantly associated with a poorer OS. Further PSM analysis, which aimed to cancel out the potential influence of selective bias between patients with elevated PCT or not, also demonstrated that a PCT concentration ≥ 0.39 ng/mL at POD 5 was an independent predictor for poorer survival in the matched 901 patients. Taken together, the present study revealed that an elevated PCT concentration at POD 5 significantly influenced the prognosis of GA patients undergoing radical gastrectomy. External validation and the matched cohort confirmed our findings, which offered statistical power to improve the reliability of the conclusions.

The PCT concentration is considered to be an accurate predictor for systemic inflammation, especially for the diagnosis of bacterial infections [8–11]. In patients with bacterial infection, PCT levels increased 2–4 h following bacterial stimulus and peaked at 12–24 h, with a half-life about 24 h [28]. But in patients receiving elective colorectal surgery, PCT levels reached peak at POD 2 and a subsequent decrease in the following 2 days [29]. Meanwhile, infection and chronic inflammation has long been considered to play an essential role in the development of varied types of cancers and to be involved in promoting all stages of tumorigenesis, such as tumor growth, progression and metastasis [30]. There is mounting evidence that the suppressed immunity status of cancer patients not only increases the incidence of post-operative complications, but also adversely impacts the long-term oncological outcomes.

In recent years, many studies have highlighted the predictive role of PCT on the prognosis of several cancers, such as medullary thyroid cancer, NSCLC, colorectal cancer, neuroendocrine neoplasms, and so on [12–15, 31]. In a prospective population-based study that enrolled 3322 patients [32], the baseline PCT was found to be significantly associated with all-cause mortality and cancer-related mortality in men after a median follow-up time of 16.2 years. In a retrospective study of 277 colorectal cancer patients who received surgery, the pre-operative PCT concentration was identified as a significant predictor for cancer-specific survival in stages I to III of the disease [15]. In another retrospective study consisting of 95 metastatic NSCLC patients who received immune-check point inhibitors, a baseline PCT concentration of > 0.1 mg/L was confirmed to result in significantly worse outcomes in terms of OS [33]. In contrast, Booka and colleagues [13] reported that in 105 esophageal cancer patients who underwent esophagestomy, a low PCT concentration (< 1 ng/mL) at POD 2 was an independent predictor of poorer OS. Possible explanations for the contradictory conclusions might be the inconsistency of patients inclusion criteria, varied PCT measurement times and the PCT cutoff concentration, relatively small sample sizes and the retrospective nature of the studies.

As far as we are aware, whether the PCT concentration is associated with the prognosis of GA patients has never been investigated. Therefore, we conducted the present study with a large sample size from an extended cohort of a prospective study. As described in Table 2, although lymphocyte count, NLR and PNI was considered as potential predictor for OS in univariate analysis in the training cohort, all of them lost their significance after multivariate analyses. In contrast, elevated PCT level at POD 5 was confirmed as an independent predictor for prognosis, which showed advantages comparing with other inflammation factors. In order to examine the generalizability of our findings, we further performed external validation and PSM analysis.

As shown in Table 1, the baseline characteristics differed significantly between the training and validation groups, including the values for intra-operative bleeding, tumor stage and the incidence of post-operative complications, which are well-established predictors for the prognosis of GA patients [25, 34]. External validation using the dataset with significantly different baseline characteristics ascertained the robust prediction power of PCT for OS. In addition, as described in Table 4, some important variables, such as BMI, tumor stage and post-operative complications, were significantly different among patients with elevated PCT concentrations or not before matching. In order to eliminate potential selective bias and to mimic a randomized trial, we further conducted a PSM analysis. After matching, all baseline characteristics were comparable between the two groups. Further univariate and multivariate Cox regression analyses also confirmed that an elevated PCT concentration at POD 5 was independently associated with OS in the matched cohort. Taken together, external validation and PSM analyses added additional statistical power to make our final conclusions more reliable.

Although few research groups have reported the relationship between the PCT concentration and prognosis of cancer patients, the underlying mechanism(s) has not been unequivocally clarified. Baseline PCT was generally secreted by thyroid C-cells and other neuroendocrine cells in the lung and bowel, and predominantly by the cells in the lung and bowel when inflammation occurred [12]. In the hypoxic tumor micro-environment, pro-inflammatory factors such as tumor necrosis factor-α (TNF-α) and interleuin-6 (IL-6) were produced as a result of tissue damage and/or tumor necrosis, which might further activate the systemic inflammatory response [35]. As a result, increased concentrations of serum PCT levels facilitated tumor metastasis and led to poor oncological outcomes [36]. In addition, bacterial toxins including endotoxins could directly stimulate the production of PCT in patients with developing infections, which might also weaken the immune system and facilitate tumor progression [13]. In the present study, a patient with a higher PCT concentration had a significantly worse prognosis no matter whether they developed an infection or not. Although the difference in the infection group was slightly outside a statistically significant level (*P* = 0.062), most likely due to the relatively small sample size (n = 51). Additionally, an additive effect was confirmed in patients with elevated PCT concentrations and suffering from an infection. Thus, it seemed that the adverse impact of the PCT concentration on prognosis was independent of infection. Therefore, further studies are still needed to investigate the exact underlying mechanisms.

The present study had several limitations. First, patients’ pre-operative PCT concentrations were not measured. Thus, the association between the baseline PCT concentration and prognosis, and the impact of the dynamic change of the PCT concentration before and after surgery on prognosis could not be investigated. Second, the median follow-up time of 30 months for the entire cohort was insufficient to analyze later relapses and deaths of patients. Third, patients undergoing neo-adjuvant chemotherapy were excluded from the present study. As a result, whether our findings could be used in these patients needs further external validations. Last, but by no means least, given that different laboratory examination methods used to measure PCT concentrations, the PCT concentration might differ significantly among institutions and therefore it seemed inappropriate to cite our cutoff values directly.

In conclusion, for the first time, our study has revealed that an elevated PCT concentration (≥ 0.39 ng/mL) at POD 5 was a significant predictor for poorer prognosis in GA patients following radical gastrectomy. The extended cohort from a prospective study with a large sample-size of patients, the combination use of external validation and PSM analysis improved the reliability of our final conclusions. But further studies are still needed to clarify the exact underlying mechanisms.

### Supplementary Information

Below is the link to the electronic supplementary material.

Supplementary file 1: **Figure 1. **X-tile analyses of overall survival (OS) performed using patients data to determine the optimal cut-off value for procalcitonin (PCT) level at post-operative day (POD) 3 and POD 5 **A-D **and **E-H**. The optimal cut-off value for PCT at POD 3 and POD 5 for OS were set at 0.67 ng/mL and 0.39 ng/mL, when they reached the maximum χ^2^ values of 8.8624 and 14.3769, separately **A** and **E**. In the left panels **B** and **F**, the X-axis represents all potential cut-off values from low to high (left to right) that define a low subset, whereas the Y-axis represents the cut-off values from high to low (top to bottom) that define a high subset. Red coloration of a cut-off value indicates an inverse correlation with time to recurrence, and green coloration represents direct associations. The optimal cut-off values highlighted by the black circles in the left panels are shown in the histograms of the entire cohort **C** and **G**. Kaplan-Meier curves are displayed in the right panels **D** and **H**, where blue represents the low subgroup and gray represents the high subgroup.

Supplementary file 2: **Figure 2.** Overall survival curves of the entire patients in the training cohort who underwent curative resection for stage I-III gastric cancer stratified by procalcitonin (PCT) level at post-operative day (POD) 5 and post-operative infection. (PCT low defined as < 0.39 ng/mL, PCT high defined as ≥ 0.39 ng/mL, infection (-) defined as no infection, infection (+) defined as developing post-operative infection).

Supplementary file 3: **Figure 3.** Over survival curves of the entire patients who underwent curative resection for stage I-III gastric cancer stratified by procalcitonin (PCT) level at post-operative day (POD) 5 (< 0.39 or ≥ 0.39 ng/mL) before matching **A** and after matching **B**.

Supplementary file 4: (DOC 89 KB)

## Data Availability

The data that support the findings of this study are available on reasonable request from the corresponding author.

## Abbreviations

ASA: American Society of Anesthesiologists
BMI: Body mass index
CapOx: Capecitabine and oxaliplatin combination
CI: Confidence interval
CRP: C-reactive protein
GA: Gastric adenocarcinoma
GPS: Glasgow prognostic score
HR: Hazard ratio
IL-6: Interleuin-6
NLR: The neutrophil to lymphocyte ratio
NSCLC: Non-small cell lung cancer
OS: Overall survival
PCT: Procalcitonin
PNI: Prognostic nutritional index
POD: Post-operative day
PSM: Propensity score matching
RFS: Recurrence free survival
SOX: Oxaliplatin and S-1 combination
TNF-α: Tumor necrosis factor-α
TNM: Tumor-node-metastasis
