# Supplementary figures and images for: Prognostic value of post-operative serum procalcitonin in gastric adenocarcinoma patients undergoing radical gastrectomy: propensity score matching analysis of extended cohort from a prospective bi-center study
Source: Gastric Cancer. 2023 Aug 14;26(6):1051–62. doi: 10.1007/s10120-023-01422-0 (PMC10640415; doi:10.1007/s10120-023-01422-0)

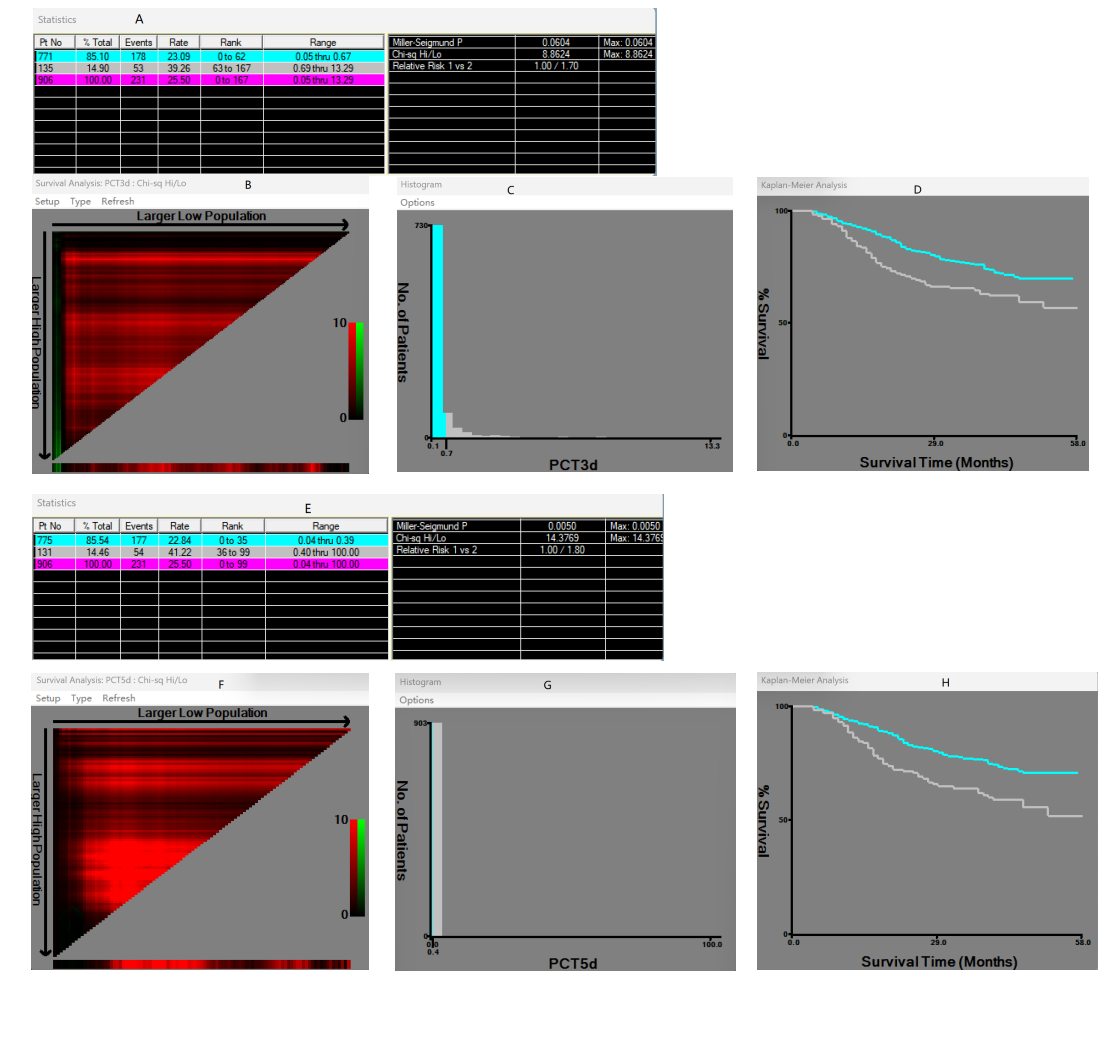

Supplement: Supplementary file 1 — Figure 1. X-tile analyses of overall survival (OS) performed using patients data to determine the optimal cut-off value for procalcitonin (PCT) level at post-operative day (POD) 3 and POD 5 A-D and E-H. The optimal cut-off value for PCT at POD 3 and POD 5 for OS were set at 0.67 ng/mL and 0.39 ng/mL, when they reached the maximum χ2 values of 8.8624 and 14.3769, separately A and E. In the left panels B and F, the X-axis represents all potential cut-off values from low to high (left to right) that define a low subset, whereas the Y-axis represents the cut-off values from high to low (top to bottom) that define a high subset. Red coloration of a cut-off value indicates an inverse correlation with time to recurrence, and green coloration represents direct associations. The optimal cut-off values highlighted by the black circles in the left panels are shown in the histograms of the entire cohort C and G. Kaplan-Meier curves are displayed in the right panels D and H, where blue represents the low subgroup and gray represents the high subgroup. [file 10120_2023_1422_MOESM1_ESM.tif]

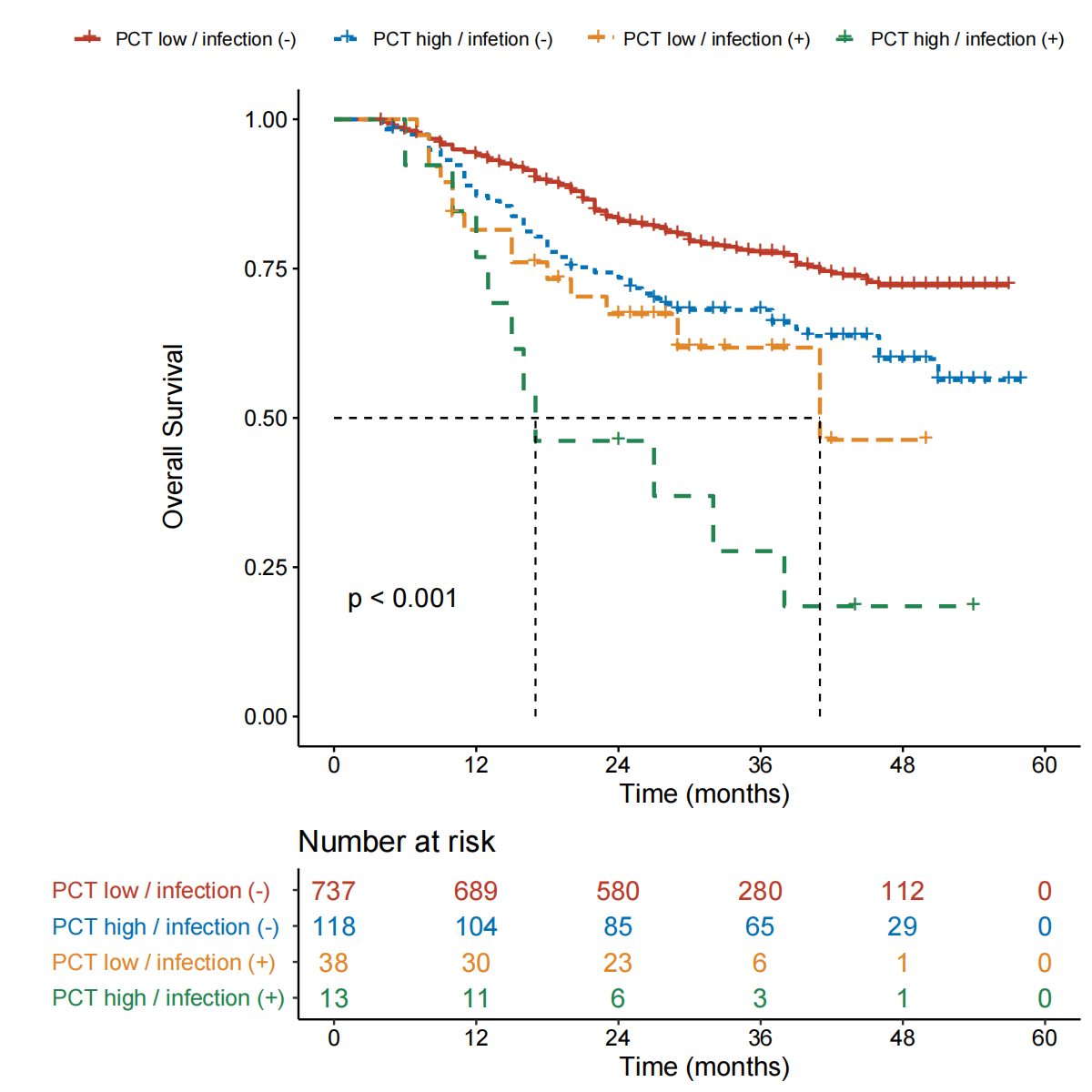

Supplement: Supplementary file 2 — Figure 2. Overall survival curves of the entire patients in the training cohort who underwent curative resection for stage I-III gastric cancer stratified by procalcitonin (PCT) level at post-operative day (POD) 5 and post-operative infection. (PCT low defined as < 0.39 ng/mL, PCT high defined as ≥ 0.39 ng/mL, infection (-) defined as no infection, infection (+) defined as developing post-operative infection). [file 10120_2023_1422_MOESM2_ESM.tif]

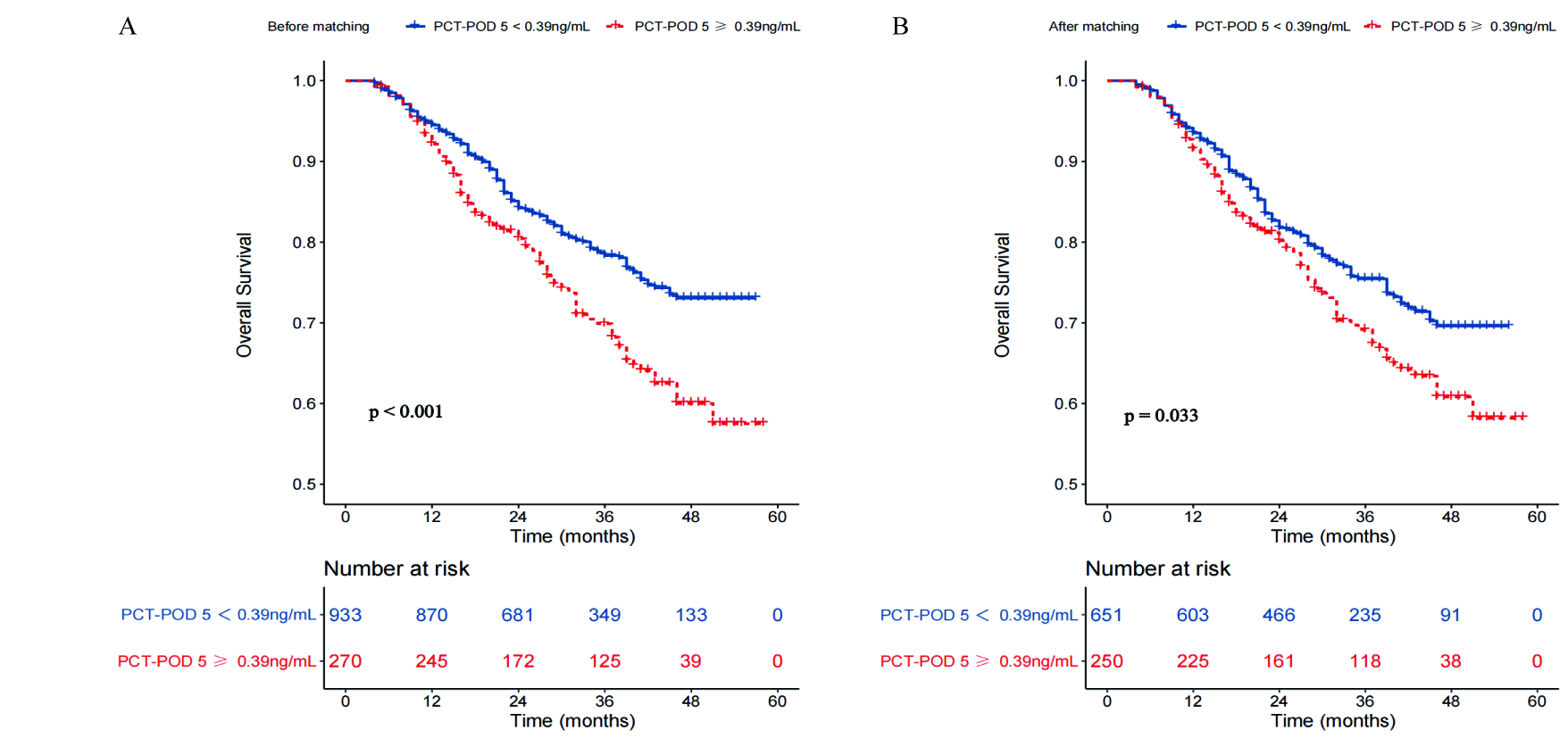

Supplement: Supplementary file 3 — Figure 3. Over survival curves of the entire patients who underwent curative resection for stage I-III gastric cancer stratified by procalcitonin (PCT) level at post-operative day (POD) 5 (< 0.39 or ≥ 0.39 ng/mL) before matching A and after matching B. [file 10120_2023_1422_MOESM3_ESM.tif]
