# Supplementary material for: Prognostic value of post-operative serum procalcitonin in gastric adenocarcinoma patients undergoing radical gastrectomy: propensity score matching analysis of extended cohort from a prospective bi-center study
Source: Gastric Cancer. 2023 Aug 14;26(6):1051–62. doi: 10.1007/s10120-023-01422-0 (PMC10640415; doi:10.1007/s10120-023-01422-0)
Supplement: Supplementary file 4 — (DOC 89 KB) [file 10120_2023_1422_MOESM4_ESM.doc]

| **Supplementary Table 1.** Univariate and multivariate analyses of prognostic factors for overall survival following radical gastrectomy of gastric adenocarcinoma in the validation cohort (n = 297) | | | | | |
| --- | --- | --- | --- | --- | --- |
| Variables | N | 3-year overall survival rate (%) | UV  *P* value | MV  HR (95% CI) | MV  *P* value |
| Gender |  |  | 0.779 |  |  |
| Male | 192 | 82.0% |  |  |  |
| Female | 105 | 78.1% |  |  |  |
| Age (years) |  |  | 0.003 |  | 0.016 |
| < 65 | 217 | 84.2% |  | Reference |  |
| ≥ 65 | 80 | 67.1% |  | 2.215 (1.159-4.231) |  |
| Body mass index (kg/m^2^) |  |  | 0.006 |  | 0.039 |
| ≥ 18.5 | 269 | 83.3% |  | Reference |  |
| < 18.5 | 28 | 60.5% |  | 2.190 (1.041-4.604) |  |
| Comorbidities |  |  | 0.308 |  |  |
| Yes | 41 | 75.9% |  |  |  |
| No | 256 | 82.0% |  |  |  |
| Hemoglobin (g/L) |  |  | 0.530 |  |  |
| ≥ 100 | 241 | 80.4% |  |  |  |
| < 100 | 56 | 78.7% |  |  |  |
| Albumin level (g/L) |  |  | 0.088 |  | 0.457 |
| ≥ 35 | 258 | 81.5% |  |  |  |
| < 35 | 39 | 66.7% |  |  |  |
| Lymphocyte count (×10 9/L) |  |  | 0.051 |  | 0.143 |
| ≥ 1.5 | 224 | 82.7% |  |  |  |
| < 1.5 | 73 | 53.0% |  |  |  |
| Intra-operative blood loss (mL) |  |  | 0.175 |  |  |
| < 300 | 267 | 79.9% |  |  |  |
| ≥ 300 | 30 | 85.4% |  |  |  |
| pTNM stage ^†^ |  |  | <0.001 |  | <0.001 |
| I | 115 | 92.2% |  | Reference |  |
| II | 77 | 87.0% |  | 1.458 (0.468-4.545) |  |
| III | 105 | 62.9% |  | 6.169 (2.384-15.959) |  |
| Post-operative complication ^‡^ |  |  | 0.507 |  |  |
| No | 222 | 80.6% |  |  |  |
| Yes | 75 | 80.9% |  |  |  |
| Procalcitonin level at post-operative day 3 (ng/mL) |  |  | 0.040 |  | 0.915 |
| < 0.67 | 61 | 86.9% |  |  |  |
| ≥ 0.67 | 236 | 76.5% |  |  |  |
| Procalcitonin level at post-operative day 5 (ng/mL) |  |  | 0.001 |  | 0.031 |
| < 0.39 | 158 | 87.2% |  | Reference |  |
| ≥ 0.39 | 139 | 73.3% |  | 2.136 (1.073-4.252) |  |
| CI, confidence interval; HR, hazard ratio; UV, univariate analysis; MV, multivariate analysis.  † Tumor stages are based on 8th edition of AJCC TNM classification.  ‡ Defined as Clavien-Dindo grade II or greater. | | | | | |
